# Tumour-infiltrated cortex participates in large-scale cognitive circuits

**DOI:** 10.1016/j.cortex.2024.01.004

**Published:** 2024-04

**Authors:** Ayan S. Mandal, Chemda Wiener, Moataz Assem, Rafael Romero-Garcia, Pedro Coelho, Alexa McDonald, Emma Woodberry, Robert C. Morris, Stephen J. Price, John Duncan, Thomas Santarius, John Suckling, Michael G. Hart, Yaara Erez

**Affiliations:** a Brain-Gene Development Lab, Department of Psychiatry, Perelman School of Medicine at the University of Pennsylvania, USA; b Brain Mapping Unit, Department of Psychiatry, University of Cambridge, UK; c Faculty of Engineering, Bar-Ilan University, Ramat-Gan, Israel; d Medical Research Council, Cognition and Brain Sciences Unit, University of Cambridge, UK; e Department of Medical Physiology and Biophysics, Instituto de Biomedicina de Sevilla (IBiS) HUVR/CSIC/Universidad de Sevilla/CIBERSAM, ISCIII, Sevilla, Spain; f Neurophys Limited, UK; g Department of Neuropsychology, Cambridge University Hospitals NHS Foundation Trust, UK; h Department of Neurosurgery, Cambridge University Hospitals NHS Foundation Trust, UK; i Division of Neurosurgery, Department of Clinical Neurosciences, University of Cambridge, UK; j Department of Experimental Psychology, University of Oxford, UK; k Department of Physiology, Development and Neuroscience, University of Cambridge, UK; l Behavioural and Clinical Neuroscience Institute, University of Cambridge, UK; m Cambridge and Peterborough NHS Foundation Trust, UK; n St George's, University of London & St George's University Hospitals NHS Foundation Trust, Institute of Molecular and Clinical Sciences, Neurosciences Research Centre, Cranmer Terrace, London, UK; o Gonda Multidisciplinary Brain Research Center, Bar-Ilan University, Ramat-Gan, Israel

**Keywords:** Glioma, Neural circuits, Electrocorticography (ECOG), Executive function, Functional connectivity, fMRI, Brain tumour

## Abstract

The extent to which tumour-infiltrated brain tissue contributes to cognitive function remains unclear. We tested the hypothesis that cortical tissue infiltrated by diffuse gliomas participates in large-scale cognitive circuits using a unique combination of intracranial electrocorticography (ECoG) and resting-state functional magnetic resonance (fMRI) imaging in four patients. We also assessed the relationship between functional connectivity with tumour-infiltrated tissue and long-term cognitive outcomes in a larger, overlapping cohort of 17 patients. We observed significant task-related high gamma (70–250 Hz) power modulations in tumour-infiltrated cortex in response to increased cognitive effort (i.e., switch counting compared to simple counting), implying preserved functionality of neoplastic tissue for complex tasks probing executive function. We found that tumour locations corresponding to task-responsive electrodes exhibited functional connectivity patterns that significantly co-localised with canonical brain networks implicated in executive function. Specifically, we discovered that tumour-infiltrated cortex with larger task-related high gamma power modulations tended to be more functionally connected to the dorsal attention network (DAN). Finally, we demonstrated that tumour-DAN connectivity is evident across a larger cohort of patients with gliomas and that it relates to long-term postsurgical outcomes in goal-directed attention. Overall, this study contributes convergent fMRI-ECoG evidence that tumour-infiltrated cortex participates in large-scale neurocognitive circuits that support executive function in health. These findings underscore the potential clinical utility of mapping large-scale connectivity of tumour-infiltrated tissue in the care of patients with diffuse gliomas.

## 1 Introduction

Diffuse gliomas are slowly progressing brain tumours that infiltrate nearby healthy tissue (Santarius et al., 2019). Recent evidence has indicated that gliomas integrate within their surrounding neural environment (Venkatesh et al., 2017, 2019; Venkatesh & Monje, 2017). It has been shown that glioma cells can form synapses with neurons (Venkataramani et al., 2019) and that neuronal signalling can trigger tumour proliferation (Pan et al., 2021). Despite evidence for interference of the tumour with neural circuits (Aabedi et al., 2021), clinically it is known that at least to some extent glioma-infiltrated brain tissue can remain involved in motor and language functions (Young et al., 2020), evidence that motivates the use of intraoperative mapping to preserve functionality (Duffau, 2011). However, it is unknown whether tumour-infiltrated tissue participates in whole-brain, large-scale distributed neural circuits involved in behaviour and cognition.

To identify functionality in the surroundings of the tumour, brain tissue is typically mapped intraoperatively using direct electrical stimulation (DES), usually focussing on motor and language-related functions. However, for some functions it is more challenging to use DES, for example executive functions, a collection of cognitive processes involved in goal-directed behaviour such as attention, planning and task-switching. These functions are more complicated to map using DES because they are supported by distributed frontal and parietal regions (Duncan & Owen, 2000; Shashidhara, Spronkers, & Erez, 2020) rather than any single, confined area. Additional information about functionality of tissue in the vicinity of the tumour may be obtained by recording brain activity directly from the surface of the brain during task performance using intracranial electrocorticography (ECoG) (Aabedi et al., 2021; Erez et al., 2021). While the use of DES for mapping executive functions is scarce (Puglisi et al., 2018, 2019; Wager et al., 2013), we have recently employed ECoG to map peritumoural brain regions responsive to a task probing executive function (Assem et al., 2023; Erez et al., 2021), further supported by another case report (Mandonnet et al., 2020). Nevertheless, while DES and ECoG are useful for mapping functional regions locally, they do not provide information about the extent to which tumour-infiltrated tissue participates in distributed, whole-brain, neural circuits associated with the tested function, as can be measured with functional connectivity using fMRI data. This information is critical especially for cognitive skills such as executive functions as they are supported by coordinated activity in distributed regions in the frontal and parietal cortex (Duncan & Owen, 2000; Fedorenko et al., 2013).

Assessing functional connectivity within glioma-infiltrated tissue has been traditionally controversial given that tumours are known to interfere with neurovascular coupling, upon which the fMRI BOLD (blood-oxygenation level dependent) signal depends (Pak et al., 2017). However, it has been recently demonstrated that functional connectivity can be routinely identified within glioma-infiltrated tissue, further supporting findings from other methods like DES in establishing functionality (Cui et al., 2022; Daniel et al., 2021). While gliomas have been demonstrated to commonly localize to functional network hubs (Mandal et al., 2020, 2021, 2023; Numan et al., 2022; Romero-Garcia, Mandal, et al., 2022), it remains unclear whether glioma-infiltrated tissue participates in large-scale neural circuits important for cognitive function.

**Fig. 1:**
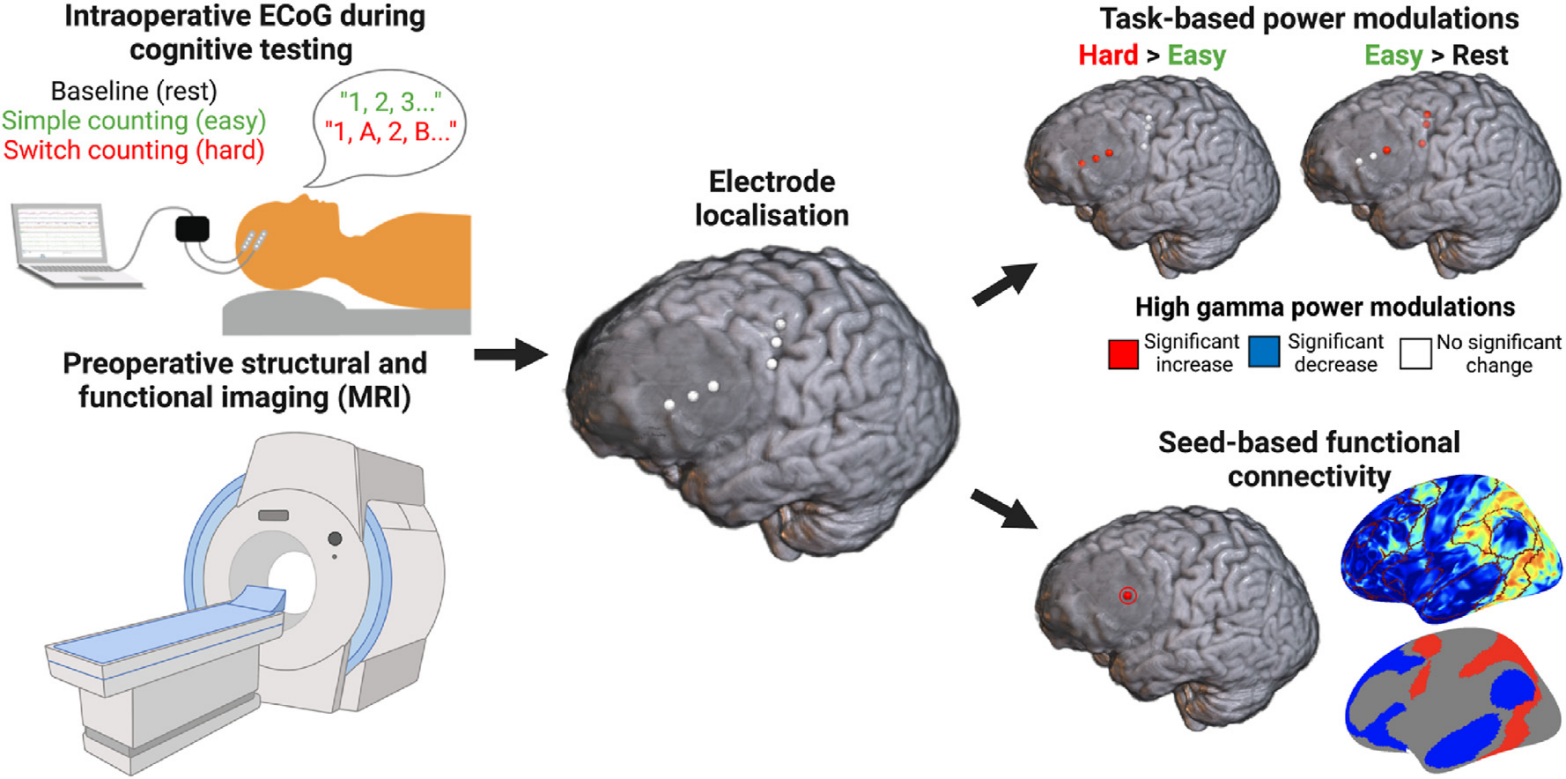
Schematic overview of the study. Participants underwent preoperative neuroimaging (bottom left) and intraoperative electrocorticography (ECoG) (top left) during three conditions (rest, simple counting, and switch counting). Locations of electrodes on tumour-infiltrated (blurred area) versus non-infiltrated tissue were then localized on the preoperative structural scan (middle). Signal change in high gamma power between task conditions for each electrode was assessed (top right), reflecting task-related neural activity. Finally, whole-brain seed-based functional connectivity analyses were performed with the tumour electrode locations as seeds (bottom right, with significant network connectivity in red and blue), linking data across modalities. The correspondence between each resulting functional connectivity map and the topography of canonical functional networks was assessed for significance using a spin test (bottom right) (Alexander-Bloch et al., 2018).

Here, we tested the hypothesis that cortical tissue infiltrated by glioma participates in large-scale cognitive circuits using a unique ECoG-fMRI dataset (Fig. 1). We further extended our findings in an overlapping, larger cohort using fMRI data of tumour-infiltrated tissue and longitudinal cognitive assessment. We used ECoG to identify task-related activity in the form of power modulations in response to increased cognitive demand, probing executive function. We expected to find task responses in parts of tumour-infiltrated frontal cortex, as well as functional connectivity between task-responsive tumour locations and canonical functional networks associated with executive function.

## 2 Materials and methods

### 2.1 Patient recruitment

We initially recruited 18 patients suspected of a diffuse low-grade glioma and scheduled to undergo an awake craniotomy at the Department of Neurosurgery at Cambridge University Hospital. All participants underwent surgery using a protocol utilising total intravenous anaesthesia with propofol and remifentanil, and were awakened for the intraoperative testing. One patient dropped out of the study due to difficulty tolerating the MRI environment. For this study, we conducted two sets of analyses on (1) a cohort where both ECoG and fMRI data of tumour-infiltrated cortex were available in the same participants (“ECoG-fMRI Cohort”), as well as (2) an overlapping cohort of participants where fMRI data of tumour-infiltrated cortex were available (“fMRI Cohort”).

Out of the initial 17 patients, an electrode strip was noted during the surgery to be placed upon tumour-infiltrated cortex for five participants. One of these participants was eventually excluded since electrode co-registration with MRI did not confirm placement of any of their electrodes on tumour-infiltrated cortex. Therefore, data from four participants were included and used for further analysis in the ECoG-fMRI Cohort. Demographic and pathological information of the patients is included in Table 1. All participants gave written informed consent to participate and were aware that the research would not affect their clinical care before, during, or after the surgery. Due to clinical ethical considerations and in accordance with the ethics approval for the study by the East of England–Cambridge Central Research Ethics Committee (REC reference 16/EE/0151), the data cannot be shared or become publicly available. While patient data cannot be shared, code used to reproduce the main figures of the manuscript are available in the following repository (https://github.com/ErezNeuroLab/Glioma-participation-in-cognitive-circuits.git). No part of the study procedures or analyses was pre-registered prior to the research being conducted. We report how we determined our sample size, all data exclusions (if any), all inclusion/exclusion criteria, whether inclusion/exclusion criteria were established prior to data analysis, all manipulations, and all measures in the study.

**Table 1:** Demographic and pathological variables for each participant.

Patient ID	Age	Sex	Hemisphere	Tumour location	Grade	Pathology	Molecular genetics	Treatment	Notes
**ECoG-fMRI Cohort**									
Patient 1	41	Female	Left	Frontal	II	Oligodendroglioma	IDH-mut 1p19q-codeletion	Chemo-RT	
Patient 2	38	Female	Right	Frontal	II	Oligodendroglioma	IDH-mut 1p19q-codeletion	Chemo-RT	
Patient 3	29	Male	Left	Frontal	III	Astrocytoma	IDH-mut 1p19q-non-codeleted	Chemo-RT	
Patient 4	32	Male	Left	Temporal	III	Astrocytoma	IDH-mut	NA	Limited performance on switch counting, therefore excluded from hard > easy contrast
**fMRI Cohort**									
Patient 5	32	Male	Right	Insula	II	Glioblastoma	IDH-wt	Chemo-RT	Missed all post-operative cognitive assessments
Patient 6	26	Male	Left	Insula	IV	Astrocytoma	IDH-mut 1p19q-non-codeleted	60Gym concomitant not adjuvant temozolomide	
Patient 7	49	Female	Right	Insula	II	Oligodendroglioma	IDH-mut 1p19q codeletion	Observation	
Patient 8	55	Female	Left	Frontal	II	Oligodendroglioma	IDH-mut 1p19q codeletion	Observation	
Patient 9	22	Female	Left	Frontal	I	Ganglioglioma	IDH-wt	Observation	
Patient 10	29	Male	Right	Frontal	III	Astrocytoma	IDH-mut 1p19q codeletion	Chemo-RT	
Patient 11	29	Male	Right	Frontal	II	Astrocytoma	IDH-mut 1p19q codeletion	Chemo-RT	
Patient 12	50	Male	Left	Temporal	IV	Glioblastoma	IDH-wt	Chemo-RT	
Patient 13	33	Female	Left	Temporal	III	Astrocytoma	IDH-mut 1p19q non-codeleted	59.6/33, temozolomide	Did not complete pre-operative Hearts Cancellation task
Patient 14	27	Female	Left	Temporal	I	Ganglioglioma	IDH-wt	Observation	
Patient 15	56	Female	Left	Temporal	NA	Astrocytoma	NA	Chemo-RT	
Patient 16	27	Male	Left	Frontal	IV	Glioblastoma	IDH-wt	Chemo-RT	
Patient 17	30	Male	Left	Frontal	III	Astrocytoma	IDH-mut	Chemo-RT	

### 2.2 Behavioural tasks

Patients were introduced to the tasks during standard pre-operative visits as well as a research-dedicated pre-operative assessment. Testing was performed during the surgery and following the craniotomy, just after the patient had been awakened and prior to tumour resection. ECoG recordings were obtained during a baseline condition, and during performance of two cognitive tasks. In the baseline (rest) condition, patients were asked to stay calm and remain silent for 2–3 min. The two cognitive tasks were: simple counting (1, 2, 3, up to 20; easy) and switch counting (1, A, 2, B, 3, C, up to 20; hard). These tasks were chosen to match protocols from fMRI studies probing executive function regions with an increased cognitive demand manipulation, as has been consistently and robustly demonstrated in multiple neuroimaging studies (Assem et al., 2023; Erez et al., 2021; Fedorenko et al., 2013). Additionally, the switch counting task included a task switching element, similarly to the widely used Trail Making Test. Each task condition was repeated for 2–5 trials based on each patient's ability and time constraints during the surgery. Trial onset and offset markers were recorded manually on the acquisition system. One participant (Patient 4) was not able to perform the switch counting task correctly, instead uttering “1, A, 2, B, 3, B, 4, B …”. Therefore, these switch counting trials were instead labelled as simple counting, and the switch counting versus simple counting (hard > easy) contrast was not evaluated for this participant.

### 2.3 MRI acquisition and pre-processing

MRI data were obtained pre-operatively using a Siemens Magnetom Prisma-fit 3 T MRI scanner and 16-channel receive-only head coil (Siemens AG, Erlangen, Germany). Structural anatomic images were acquired using a T1-weighted (T1w) MPRAGE sequence (FOV 256 mm × 240 mm × 176 mm; voxel size 1 mm isotropic; TR 2300 msec; TE 2.98 msec; flip angle (FA) 9°). During the same scanning session, we also acquired resting-state (eyes closed) fMRI data with a BOLD-sensitive sequence: TR = 1060 msec, TE = 30 msec, Acceleration factor = 4, FA = 74°, 2 mm isotropic resolution, FOV = 192 × 192 mm^2^, and acquisition time of 9 min and 10 sec. fMRI pre-processing was performed using Independent Component Analysis (ICA) in FSL MELODIC, implemented to remove components contributing noise (Jenkinson et al., 2012). Additionally, we performed slice timing correction, bias field correction, rigid body motion correction, grand mean scaling, and smoothing to 5-mm ﬁxed-width half-maximum.

Masks of the tumour were drawn on the pre-operative scan using a semi-automated procedure. First, an experienced neurosurgeon (MGH) manually delineated the tumour on the pre-operative T1w image slices for each participant. Further refinement of each mask was performed using the Unified Segmentation with Lesion toolbox (https://github.com/CyclotronResearchCentre/USwithLesion) that accounts for lesion distortion by adding a subject-specific probability map before spatially warping to a reference space where tissue probability maps are predefined (Lommers et al., 2019).

### 2.4 Electrode localisation

The extent of craniotomy for each patient was determined purely by clinical considerations to allow for the tumour resection. Based on the craniotomy size and location, one to three electrode strips with four electrodes each were placed on the cortical surface. For the most part, electrode strips were placed further away from the tumour in regions judged by the neurosurgeon and later confirmed on MRI scans to be healthy, but for some participants, one strip was placed on tumour-infiltrated tissue and data from these participants were included in this study. Two types of electrode strips were used, with electrode diameter either being 5 mm (MS04R-IP10X-0JH, Ad-Tech, Medical Instruments corporation, WI, USA) or 3 mm (CORTAC 2111-04-081, PMT Corporation, MN, USA). For both types, electrodes were spaced 10 mm centre to centre.

Electrode locations for Patients 2–4 were determined using an automated method with a probe linked to a stereotactic neuronavigation system (StealthStation® S7® System, Medtronic, Inc, 24 Louisville, CO, USA). For Patient 1, locations were determined using a semi-manual grid method using intraoperative photographs and a grid-like delineation of cortical sulci and gyri due to the available localisation information. Both methods are detailed below.

(1) *Stereotactic neuronavigation*: Coordinates were recorded from the centre of each electrode during surgery using the neuronavigation system. The coordinates were then registered to the patient's preoperative T1w scan. The coordinates were further shifted to correct for electrode displacements due to brain shifts following the craniotomy.
(2) *The grid method*: This method is identical to the protocol described in Assem et al. (2023) and Erez et al. (2021), except for that the electrodes were mapped onto the participant's T1w scan instead of the MNI template. (a) Visible major sulci were delineated on the intraoperative photographs: precentral sulcus, sylvian fissure, inferior and superior frontal sulci. Spaces between these sulci were populated by vertical lines (1.5 cm apart) to create a grid-like structure. (b) A grid was created in the same way on a 3D render of the participant's preoperative, skull-stripped T1w scan, visualised using MRIcroMTL (https://www.nitrc.org/). (c) Voxel coordinates were manually extracted by marking the approximate locations on the grid from the 3D render corresponding to the electrode locations from the grids drawn on the intraoperative photographs.

For both methods, electrode displacements were then corrected for brain shifts by back-projecting the electrode locations onto the cortical surface along the local norm vector(Hermes et al., 2010) implemented in the fieldtrip (v20160629) protocol for human intracranial data (Stolk et al., 2018). Finally, electrode locations were mapped to the nearest grey matter or tumour voxel, using a nearest neighbour approach. This was done to ensure that seeds placed around the electrode locations would capture BOLD signal from cortical tissue. When a given coordinate was equidistant to two or more cortical voxels, the coordinate which ensured electrode spacing closest to 10 mm was chosen.

After electrode localisation, we constructed 2.5-mm radius spheres (to match the largest electrode diameter) around each voxel coordinate corresponding to tumour-infiltrated electrodes. We confirmed whether the electrodes were placed on the tumour-infiltrated tissue, as the surgical notes implied, by determining whether these spheres overlapped with the previously delineated tumour mask. Electrode placement on the tumour was confirmed for all electrodes from the four patients included in the ECoG-fMRI Cohort (Supplementary Fig. 1).

### 2.5 Electrophysiological data acquisition and analysis

Electrophysiological data were acquired using a 32-channel amplifier (Medtronic Xomed, Jacksonville, FS, USA) sampled at 10 KHz. Potential sources of electrical noise were identified and repositioned during surgery to avoid signal contamination. The data were recorded using dedicated channels on the acquisition system and two Butterworth online filters were applied: a high-pass filter at 1 Hz and a low-pass filter at 1500 Hz. A ground needle electrode was connected to the deltoid muscle. The electrodes were referenced to a mid-frontal (Fz) spiral scalp EEG electrode.

Data were analysed offline using custom MATLAB scripts and EEGLAB (v13.6.5b). The data were downsampled to 2 kHz then re-referenced using a bipolar scheme. The bipolar scheme was chosen to detect any activity changes with high spatial resolution as well as to avoid contamination of high frequency signals by scalp muscle artifacts detected by the Fz electrode. This involved re-referencing each electrode using an adjacent electrode on the same strip (i.e., 1 minus 2, 2 minus 3, 3 minus 4). As a result of the bipolar scheme, data from three electrodes from each strip were used for further analysis.

Line noise was removed by applying a notch filter at 50 Hz and its harmonics. Notch filtering was also applied at 79 Hz and its harmonics to remove additional noise observed in the data, likely due to equipment in the surgical theatre. Data were then bandpass filtered to extract activity in the high gamma (HG, 70–250 Hz) range. Instantaneous power of the timeseries was calculated by squaring the absolute amplitude envelope of the Hilbert transformed data. We focused on high gamma power given prior evidence of the concordance of this frequency band with BOLD signal and neuronal activity associated with cognitive processing (Hacker et al., 2017; Haufe et al., 2018), as well as evidence linking increase in HG activity following increased cognitive demand to executive function (Assem et al., 2023; Erez et al., 2021). An example of a HG timeseries is shown in Supplementary Fig. 2.

The power timeseries data were then segmented into the appropriate conditions and trials. Because trial onset and offset markers were manually recorded, two seconds from the beginning and end of the rest trial and one second from each task trial were excluded to account for error related to human reaction time. For the switch counting trials, a further three seconds were excluded from the beginning of each trial to discard the initial easy phase of this task (1, A, 2, B, 3, C).

To link neural activity to certain cognitive processes, we calculated the percentage of signal change in high gamma power for two task contrasts: hard > easy and easy > rest. The hard > easy contrast was specifically designed to identify recruited areas involved in executive function, as the conditions of this contrast were closely matched to prior studies probing such (Assem et al., 2023; Erez et al., 2021; Fedorenko et al., 2013). HG signal changes for the easy > rest contrast were thought to reflect task-responsive regions more generally, as well as regions responsible for speech production. We computed a percentage of signal change for each contrast by first calculating the average power for each condition across all trials and time points. For each contrast (hard > easy, easy > rest), the percentage of signal change was then computed as: [(power in condition 1/power in condition 2) − 1]∗100.

A permutation testing approach was used to statistically test for power modulations in each electrode while controlling for temporal autocorrelation of the signal (Erez et al., 2021). For each electrode and contrast, the instantaneous power timeseries of all task trials from both conditions in the contrast were concatenated serially to form a loop. To close the loop, the end of the last trial was joined to the beginning of the first trial. All trial onset and offset markers were then shifted using the same randomly generated jitter, allowing the power timeseries values to “rotate” along the data loop. This rotation approach was used to generate surrogate power data while preserving trial lengths and the temporal autocorrelations in the data. After the rotation, the mean power (for each condition) and percentage of signal changes (for the conditions in the contrast) was computed based on the new trial markers. This procedure was repeated 100,000 times with a different random jitter for each permutation to create a surrogate distribution against which two-tailed statistical significance (alpha = .05) was calculated for each contrast within each electrode. Significance testing was performed for both tumour-infiltrated and healthy-appearing electrodes to provide context for task-related power modulations in tumour-infiltrated cortex.

### 2.6 Seed-based functional connectivity analysis

For each electrode placed on tumour, we conducted seed-based functional connectivity analysis to determine whether the corresponding cortical tissue was functionally coupled to canonical resting state networks. Using FSL software (Jenkinson et al., 2012), the previously mentioned 2.5 mm radius spheres placed around each tumour electrode coordinate were first masked such that they covered only brain parenchyma, then mapped into the space of each participant's functional scan. Next, the mean BOLD timeseries of voxels within the seed was calculated, then cross-correlated (Pearson's) with the BOLD timeseries of each other brain voxel. A Fisher's R to Z transformation was performed on the resulting map, which was then smoothed at 5-mm full-width half maximum. Finally, the seed-based functional connectivity map was mapped back onto the space of each participant's native structural scan.

The volumetric seed-based functional connectivity maps were then projected onto surface vertices for the purposes of visualisation and statistical inference of canonical network correspondence. Using FreeSurfer reconstructions of each participant (Fischl, 2012), as well as Connectome Workbench code (Glasser et al., 2013), each connectivity map was mapped onto fsaverage (∼164k vertices) using nonlinear surface-based registration. Each map was visualised with the boundaries of the canonical Yeo 7-network parcellation outlined on the surface. This canonical 7-network parcellation was constructed previously by employing a clustering approach to rs-fMRI data from 1000 college-aged individuals (Yeo et al., 2011).

To determine whether the seed-based functional connectivity maps significantly co-localised with specific canonical functional networks, we conducted the spin test (Alexander-Bloch et al., 2018). This approach conducts a nonparametric permutation test to infer statistical significance while controlling for spatial autocorrelation. First, the empirical median connectivity values were calculated within each canonical functional network. These values were evaluated for significance by comparing to a distribution of analogous Z values computed from randomly-rotated surrogate maps. The surrogate maps were generated by projecting the original map onto the fsaverage sphere, randomly rotating the sphere, then projecting back onto the pial surface. Median connectivity values were then computed for each canonical network in the surrogate data. We generated 10,000 surrogate maps for each seed-based functional connectivity map. The percentile of median empirical connectivity within each network compared to the null distribution of each network's median connectivity values was then used to estimate two-tailed *p*-values (alpha = .05), indicating which networks were significantly over-represented or under-represented in each connectivity map (i.e., connected to the seed more/less than expected by chance).

### 2.7 Associations between task-related power modulations and functional network correspondence

Next, we were interested in the relationship between task-based power modulations derived from ECoG with the functional connectivity maps derived from rs-fMRI in tumour-infiltrated cortex. Using the *lme4* package in R, we performed 14 (2 ECoG contrasts × 7 fMRI networks) linear mixed effects models relating median functional connectivity within each canonical network with percentage of signal changes from each task contrast. Percentage of signal change was the response variable, while functional connectivity and participant ID were modelled as the fixed and random effects, respectively. Statistical significance of the resulting mixed effects models was evaluated using a Type II Wald chi-square test computed using the *car* package in R. To determine the specificity of the results to the high-gamma power band, we also performed supplementary analyses using the percentage signal change of the hard > easy contrast for the alpha, delta, beta, and gamma frequency bands.

### 2.8 Relationship of tumour-DAN connectivity with tumour location and long-term cognitive outcomes

Finally, we sought to extend the findings from our ECoG-fMRI Cohort (n = 4) to the larger fMRI Cohort (n = 17) by unpacking the relationship of dorsal attention network (DAN) connectivity to tumour-infiltrated tissue (tumour-DAN connectivity) with tumour location and long-term cognitive outcomes. First, we constructed seed-based connectivity maps of tumour-infiltrated tissue for each participant in the fMRI Cohort by calculating Pearson's correlations between each voxel in the tumour mask and the DAN. Voxels where the DAN and tumour mask overlapped were excluded, and the extent of overlap was used as a covariate in subsequent analyses. These seed-based connectivity maps were thresholded based on the significance of the Pearson's correlation subjected to Bonferroni correction (family-wise error rate (FWER) < .05). To determine the relationship between tumour location and tumour-DAN connectivity, we constructed a linear model with mean tumour-DAN connectivity as the response variable, and tumour location, hemisphere, overlap with the DAN, age, gender, and antiseizure medication used as predictors.

We also tested the relationship between tumour-DAN connectivity and measures of goal-directed attention, an essential component of executive function that we hypothesized would relate to DAN connectivity. Patients in this study were administered a battery of cognitive assessments known as OCS-Bridge pre-operatively, as well as on follow-up assessments post-operatively prior to discharge, at 3 months, and at 12 months (Romero-Garcia, Owen, et al., 2022; https://www.ocs-bridge.com/). Some patients missed various assessments due to logistical issues. We focused on two measures of attention: (1) overall accuracy on the Hearts Cancellation task and (2) overall performance on the SALT Sustained and Spatial Attention task. For the Heart Cancellation task, patients were asked to scan through a page of hearts and cross out the ones that were drawn completely within a three-minute time constraint. In the SALT Sustained and Spatial Attention Task, participants were asked to monitor a digital lighthouse on a tablet and accurately count the number of times it flashed. These measures underwent Z-score normalization based on the performance of healthy participants (Romero-Garcia, Owen, et al., 2022). For each task, we constructed a linear model where performance on the latest follow-up assessment was the response variable, and pre-operative performance, pre-operative tumour-DAN connectivity, tumour location, hemisphere, age, gender, and timepoint of the latest assessment were predictors. Categorical variables were included in the model using dummy coding. As a sensitivity analysis, we also tested the same linear model after excluding participants without a three month or greater follow-up assessment.

## 3 Results

### 3.1 Task-related power modulations in tumour-infiltrated cortical tissue

ECoG recordings were obtained from patients intraoperatively while they were awake during three tasks with increasing cognitive demand: rest (baseline), simple counting (easy), and switch counting (hard). We calculated the percentage of signal change of high gamma (70–250 Hz) power corresponding to two task contrasts (hard > easy; easy > rest) in electrodes placed on tumour-infiltrated and non-lesioned tissue (Fig. 1). Based on our previous findings, high gamma power increases in the hard > easy contrast were considered as reflecting areas associated with executive function (Assem et al., 2023; Erez et al., 2021), whereas power increases in the easy > rest contrast were interpreted as reflecting cognitive processing more generally as well as speech production (Basilakos et al., 2018). The focus of the study is on electrodes placed on tumour-infiltrated tissue, and we show results for the electrodes that were placed on non-lesioned cortex in the same patients for reference.

**Fig. 2:**
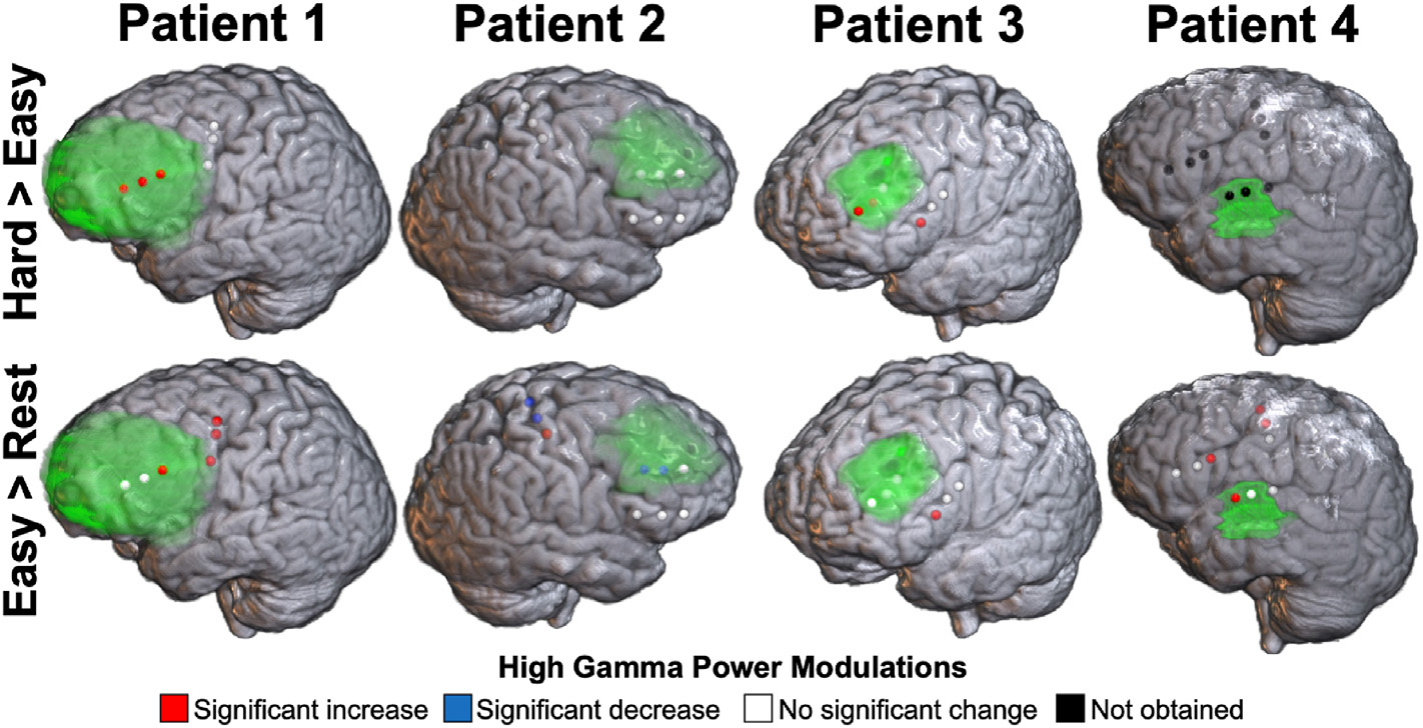
Task-related high gamma power modulations in tumour-infiltrated and non-lesioned cortical tissue. Each sphere is an electrode, coloured according to the change in high gamma power for each of the two contrasts. The hard > easy contrast was not evaluated for Patient 4 because of limited performance on the switch counting task. The tumour corresponds to the blurred green regions in the frontal cortex for Patients 1–3, and the superior temporal cortex for Patient 4.

Fig. 2 shows each electrode rendered on each participant's structural scan, colour-coded by significance of high gamma power changes for each of the contrasts. We observed significant task-related power modulations in at least one electrode for each electrode strip placed on tumour-infiltrated tissue. For two of the three participants with frontal tumours (Patient 1 and Patient 3), significantly elevated high gamma power changes were observed for the hard > easy contrast, reflecting involvement in executive function (Erez et al., 2021). One electrode on tumour-infiltrated tissue for Patient 1 also exhibited significant high gamma increases during simple counting compared to rest, an increase that may relate to speech articulation given the proximity of this electrode to the inferior frontal gyrus. Interestingly, the other participant with a frontal tumour (Patient 2) exhibited significant high gamma power decreases for the easy > rest contrast. The participant with a tumour in the temporal lobe (Patient 4) exhibited significant high gamma increases for the easy > rest contrast, possibly reflecting speech articulation which was also localized to the superior temporal gyrus in a recent fMRI study (Basilakos et al., 2018).

### 3.2 Correspondence between task-related high gamma power modulations and functional network connectivity

**Fig. 3:**
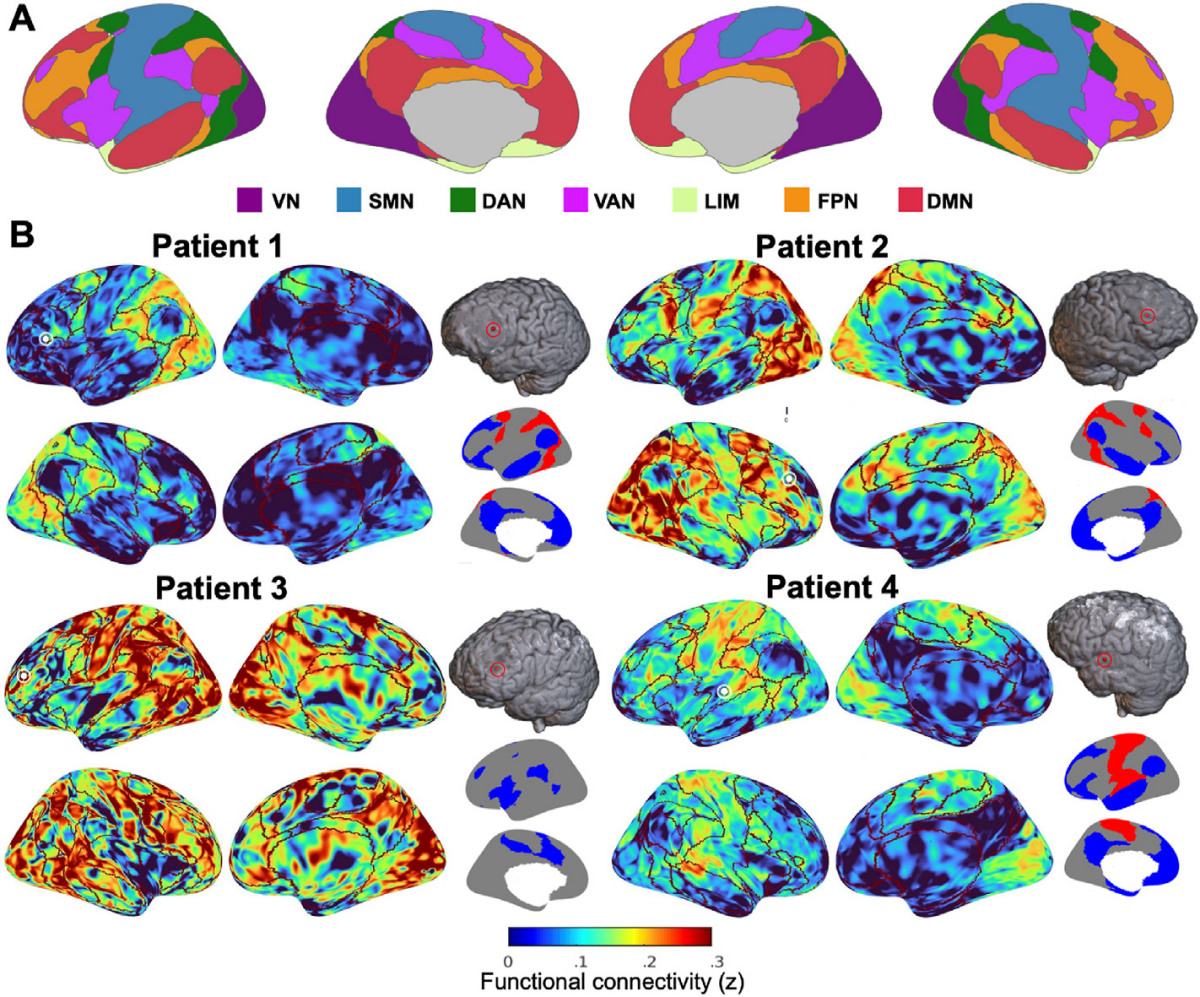
Seed-based functional connectivity of task-responsive tumour-infiltrated electrode locations. (A) Visualization of the canonical 7-network parcellation, estimated based on functional connectivity of 1000 individual (Yeo et al., 2011). (B) Examples of seed-based connectivity maps from the tumour electrode location with the largest high gamma power increase for the hard > easy contrast for each participant (except for Patient 4, for whom the easy > rest contrast was used). Boundaries of the canonical networks are outlined in dark red. Seed location is shown in the top right relative to each set of surface images (red circled sphere), as well as on the surface images themselves (white circled sphere). The canonical networks significantly overrepresented (red) and underrepresented (blue) in the functional connectivity maps are shown on the bottom right (only one hemisphere shown for ease of visualization). VN = visual network; SMN = sensorimotor network; DAN = dorsal attention network; VAN = ventral attention network; LIM = limbic network; FPN = fronto-parietal network; DMN = default mode network.

Next, we asked whether task-responsive tumour-infiltrated areas also participate in large-scale brain networks. Based on resting-state fMRI data of each participant, we constructed seed-based functional connectivity maps between each tumour electrode location and every voxel in the brain. We then compared these maps to canonical functional network maps derived previously from 1000 healthy individuals (Yeo et al., 2011) (Fig. 3A) to determine if the topology of functional connectivity from tumour-infiltrated areas retained features of healthy brain organization. Seed-based connectivity maps from the electrodes with the most elevated task-related power modulations for the hard > easy contrast for each participant (except for Patient 4, for whom the easy > rest contrast was used) are shown in Fig. 3B. Notably, the most elevated power modulation for Patient 2 was not statistically significant, though for the other participants it was. These maps demonstrate variations in strength of connectivity that are broadly aligned with boundaries of canonical functional networks.

To quantify these variations in connectivity strength, we statistically assessed the correspondence between canonical functional networks and seed-based connectivity. In the example task-responsive electrodes in Fig. 3, for both Patient 1 and Patient 2, the dorsal attention network was significantly over-represented whereas the default mode network was significantly under-represented in the connectivity maps. The ventral attention network was significantly under-represented in the connectivity map for Patient 3. For Patient 4, the sensorimotor network was significantly over-represented whereas the default mode network was significantly under-represented. These findings demonstrate that functional connectivity from tumour-infiltrated tissue is non-random, often corresponding to canonical brain networks observed in healthy populations. Furthermore, as demonstrated for Patients 1 and 2, over-represented networks such as the dorsal attention network corresponded to the task-related activity of the electrode, both associated with attention and executive function.

**Fig. 4:**
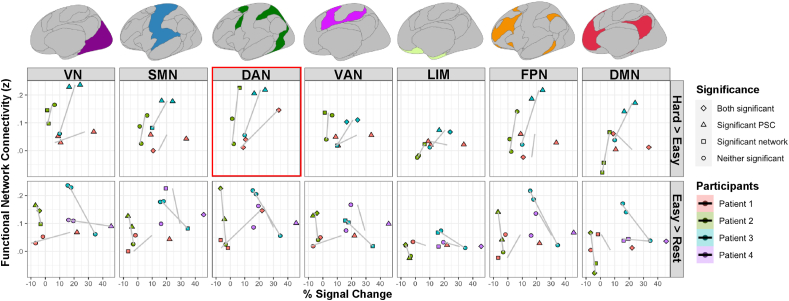
Associations between functional network connectivity and task-related power modulations in tumour-infiltrated cortex. The percentage of signal change (PSC) of each tumour electrode for each task contrast is plotted on the x-axis, against the median connectivity of that electrode location with each canonical functional network on the y-axis. Canonical functional networks are displayed above the plot with colours corresponding to the labels in Fig. 3A. Datapoints are coloured by participants. Shapes denote the significance of each individual electrode's association with the high gamma power modulation in the corresponding task contrast and network functional connectivity. Trendlines based on each individual participant's data are shown in grey. Scatterplots corresponding to a significant association between network connectivity and task-based power modulations, as assessed by a linear mixed-effects model, are outlined in red. Note that significance of connectivity values using the spin test is determined by comparison to other functional networks associated with the same electrode. Therefore, statistical significance results may be different for similar connectivity values of different electrodes. VN = visual network; SMN = sensorimotor network; DAN = dorsal attention network; VAN = ventral attention network; LIM = limbic network; FPN = fronto-parietal network; DMN = default mode network.

Next, we assessed the correspondence between high gamma power modulations and functional network connectivity across all participants and electrodes in tumour-infiltrated cortex. A significant association was observed between dorsal attention network (DAN) connectivity and high gamma power modulations for the hard > easy contrast [χ^2^(1) = 4.31; *p* = .038], i.e., increased task-related high gamma power changes in tumour-infiltrated cortex were associated with increased functional connectivity of these electrodes with the DAN (Fig. 4). No other associations between high gamma power modulations and functional network correspondence were significant [χ^2^(1) < 2.42; *p* > .1]. While the study was *a priori* focused on the high gamma band based on our previous findings (Assem et al., 2023; Erez et al., 2021), results were largely similar for the delta (1–4 Hz) and gamma (30–70 Hz) frequency bands, with DAN connectivity relating to power changes for the hard > easy contrast (Supplementary Table 1).

### 3.3 Tumour-DAN connectivity is related to long-term cognitive outcomes

**Fig. 5:**
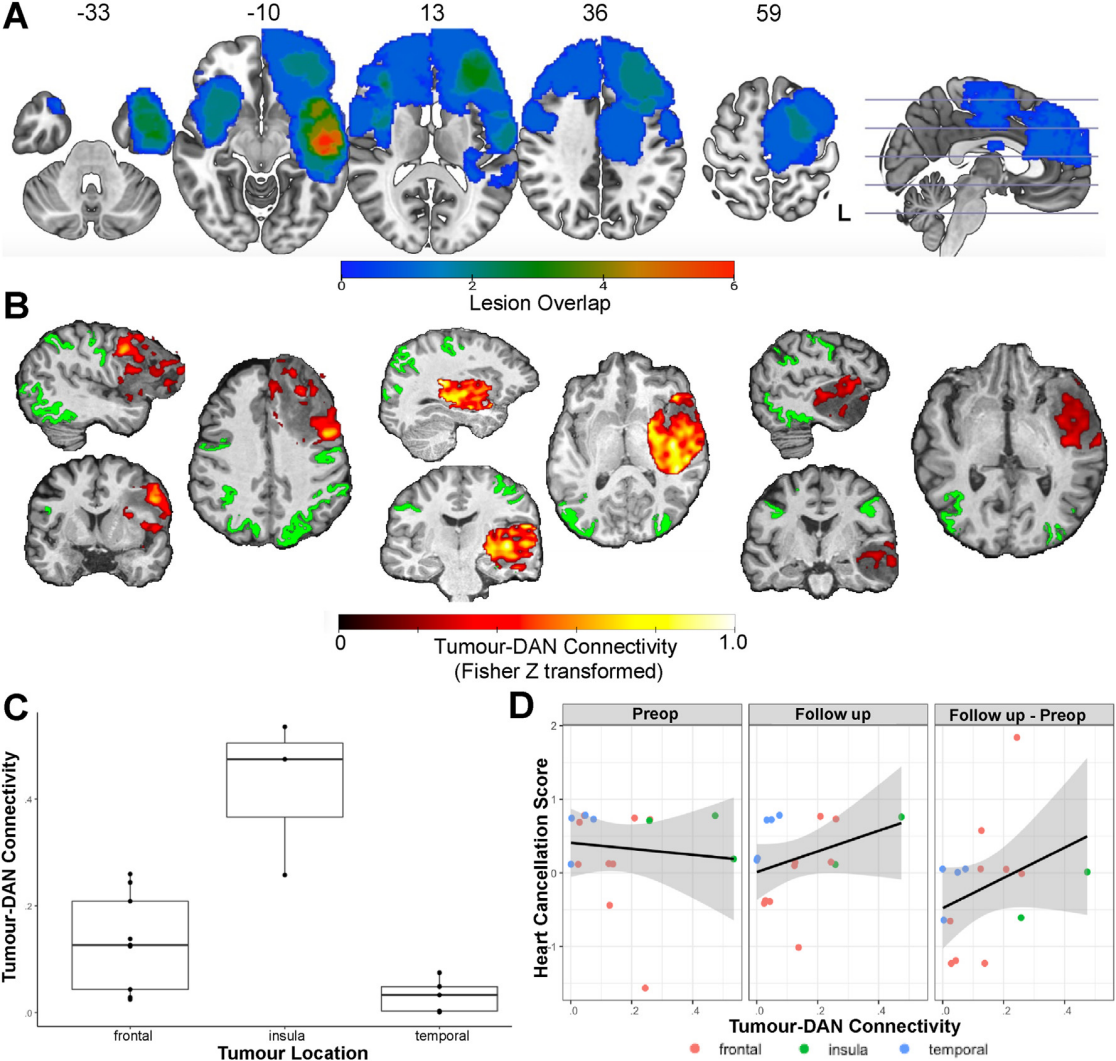
Tumour-DAN connectivity relates to tumour location and long-term cognitive outcomes. A. Lesion overlap map of the fMRI Cohort where each voxel is coloured based on the number of lesions overlapping with that voxel when registered onto the MNI template. B. Example connectivity maps of tumour-infiltrated tissue, where each voxel in the tumour mask is coloured based on the strength of its connectivity with the DAN (coloured in green). Shown are the maps with the highest average Tumour-DAN Connectivity among frontal, insular, and temporal tumours respectively. Values are thresholded based on significance of the correlation (*p* < .05, Bonferroni correction). C. Boxplot comparing average tumour-DAN connectivity across frontal, insular, and temporal tumours. Individual values for each patient are displayed as black points. D. Scatterplot of the relationship between overall accuracy on the Hearts Cancellation Task and tumour-DAN connectivity at the preop and latest follow up timepoints, as well as the difference between the follow up and preoperative scores. The preoperative and follow-up Heart Cancellation Scores were Z-score normalized using descriptive statistics from healthy participants.

To determine whether tumour-DAN connectivity is observed in a larger cohort of patients and to assess its correlates with other clinical variables of interest, we constructed seed-based connectivity maps of tumour-infiltrated tissue for each patient in the fMRI Cohort. Clinical data describing this cohort is available in Table 1, and the spatial distribution of their tumour locations is shown in Fig. 5A. Illustrative seed-based connectivity maps for the patients with the highest tumour-DAN connectivity among frontal, insular, and temporal tumours respectively are displayed in Fig. 5B. For each participant's tumour, some voxels showed significant connectivity with the DAN (*P*_FWER-corrected_ < .05), though mean connectivity across significant voxels varied considerably by tumour location (Fig. 5C). Tumour location in the insula showed significantly larger tumour-DAN connectivity compared to other tumour locations after controlling for several covariates (Table 2; standardized β = .72; *p* = .0099).

**Table 2:** Results of multiple linear regression models predicting Tumour-DAN Connectivity and long-term outcomes on tasks probing goal-directed attention.

Predictor	Standardized estimate	Standard error	T statistic	*p* Value
**Response variable = Tumour-DAN Connectivity (n = 17)**				
Intercept	NA	.15	.85	NA
**Location (insula)**	**.72**	**.09**	**3.24**	**.0099**
Location (temporal)	−.24	.07	−1.15	.28
Hemisphere (right)	9.7e-03	.085	.037	.97
Overlap with DAN	.066	1.0e-04	.32	.76
Antiseizure (yes)	.011	.082	.05	.96
Age	−.061	3.1e-03	−.29	.78
Gender (male)	.13	.069	.56	.59
**Response variable = Overall Hearts Cancellation Accuracy (follow-up) (n = 15)**				
Intercept	NA	.43	−1.6	NA
Pre-op score	.14	.13	.89	.41
**Tumour-DAN Connectivity**	**1.10**	**.96**	**4.8**	**.0049**
Location (insula)	−.36	.41	−1.4	.23
**Location (temporal)**	**.75**	**.22**	**4.2**	**.0089**
Hemisphere (right)	−.27	.26	−1.18	.29
Latest Assessment (Month 3)	.10	.22	.62	.56
Latest Assessment (Post-op)	−.41	.29	−2.2	.079
Age	.074	.0096	.37	.73
Gender (male)	.060	.19	.34	.75
**Response variable = SALT Sustained and Spatial Attention (follow-up) (n = 16)**				
Intercept	NA	1.3	.44	NA
Pre-op score	.18	.25	.49	.64
Tumour-DAN Connectivity	−.67	3.0	−1.26	.26
Location (insula)	.68	1.1	1.3	.24
Location (temporal)	−.54	.67	−1.2	.26
Hemisphere (right)	−.36	.76	−.73	.50
Latest Assessment (Month 3)	−.64	.67	−1.2	.26
Latest Assessment (Post-op)	.05	.87	.12	.91
Age	.24	.029	.54	.61
Gender (male)	−.21	.64	−.48	.65

Rows in bold text indicate significant parameters.

Finally, we sought to determine how tumour-DAN connectivity related to long-term postsurgical cognitive outcomes, specifically on tasks probing goal-directed attention, a hallmark of executive control. Tumour-DAN connectivity significantly correlated with overall accuracy on the Hearts Cancellation Task on post-operative, follow-up assessment after controlling for several covariates (Fig. 5D; Table 2; standardized β = 1.10; *p* = .0049). These results are also shown stratified by glioma subtype in Supplementary Fig. 3. Tumour location in the temporal lobe was also positively related to performance on this task (Table 2; standardized β = .75; *p* = .0089). The relationship between tumour-DAN connectivity and goal-directed attention remained significant when the analysis was restricted to patients whose latest assessment was at least 3 months after the operation (Supplementary Table 2; standardized β = 1.37; *p* = .0035). Tumour-DAN connectivity did not significantly relate with performance on the SALT Sustained and Spatial Attention Task (Table 2; standardized β = −.67; *p* = .26).

## 4 Discussion

In this study, we combined two independent modalities, ECoG and fMRI, to map regions of glioma-infiltrated cortex involved in neurocognitive circuits associated with executive functions. Our results provide evidence across multiple scales (local electrical activity, whole-brain connectivity, and cognition) that tumour-infiltrated cortex participates in large-scale cognitive networks. These findings highlight the clinical potential of mapping large-scale connectivity of tumour-infiltrated tissue in the clinical management of patients with diffuse gliomas.

Within the context of neurosurgery, the degree to which tumour-infiltrated cortex preserves functionality remains controversial, and efforts are being made to avoid the removal of critical areas and preserve functionality post-surgery (Duffau et al., 2003). Evidence from both direct electrical stimulation and electrocorticography recordings have demonstrated that some tumour-infiltrated cortical regions indeed remain eloquent and functionally active (Ganslandt et al., 2004; Ojemann et al., 1996; Schiffbauer et al., 2001) hence leading to the necessity of intraoperative mapping (Duffau et al., 2003). Our ECoG findings of increased high-gamma activity with increased cognitive demand provide further support for the functionality of cortex infiltrated by diffuse gliomas and its involvement in executive function. Previous studies provided evidence for areas associated with executive function in peri-tumoural areas using DES (Mandonnet et al., 2020; Puglisi et al., 2018, 2019; Wager et al., 2013). In some cases, these areas could be detected with DES in white matter only, and not in cortical areas (Puglisi et al., 2018, 2019). We have previously shown that high-gamma activity as recorded with ECoG from healthy tissue further away from the tumour can be used to identify areas associated with executive function (Assem et al., 2023; Erez et al., 2021). Here we further demonstrate that this neural signature of executive function is observed also in tumour-infiltrated regions, supporting the potential of using this technique for intraoperative functional mapping of executive control.

While stimulation and ECoG provide information about local areas that are necessary for these functions, little is known about whether and to what extent tumour-infiltrated areas also participate in distributed large-scale brain networks (Daniel et al., 2021). This information is important because of the essential role of distributed networks in supporting function and cognition (Bullmore & Sporns, 2009) and may have implications for post-surgery recovery and rehabilitation. Our findings demonstrate that task-responsive tumour-infiltrated areas, as measured with ECoG, are also part of large-scale distributed brain networks, as measured by rs-fMRI. While prior studies demonstrated that gliomas can hijack relatively local circuits involving language and motor function (Krishna et al., 2023; Meyer et al., 2003), our results show that tumour-infiltrated cortex participates in large-scale distributed cognitive networks important for higher-order cognition. Using ECoG, we observed activity associated with executive function in tumour-infiltrated areas. These task-related areas were functionally connected to distributed large-scale networks as measured with rs-fMRI, and in particular the DAN which has been associated with goal-directed attention, a hallmark of executive control.

While other studies have determined that functional coupling exists between tumour voxels and the rest of the brain (Daniel et al., 2021), here we demonstrate that this coupling is both topologically meaningful and relevant to cognitive outcomes. The finding that tumour locations exhibit functional connectivity patterns that significantly co-localize with canonical functional networks and are associated with task-responsive areas implies that BOLD signal in tumour reflects, at least in part, a meaningful signal of neural activity that is not totally confounded by neurovascular uncoupling. Intriguingly, we observed some electrodes in one of the patients (Patient 2) that demonstrated larger high gamma power at rest than during the simple counting task. This may potentially reflect tumour participation, specifically for these locations, in the default mode network, for which deactivations with task have been demonstrated both in fMRI and ECoG studies (Fox et al., 2018). However, our fMRI data did not support this idea, as the pre-operative functional connectivity of these electrode locations were more associated with the DAN, and DMN connectivity did not correlate negatively with percent signal change for the easy > rest contrast across participants (Fig. 4).

Our findings are consistent with recent evidence demonstrating the concordance between rs-fMRI and DES in determining tumour-infiltrated tissue involved in sensorimotor networks (Cui et al., 2022). Recent studies have also shown that intratumoural functional connectivity is prognostic of survival and cognitive performance (Daniel et al., 2021; Krishna et al., 2023; Romero-Garcia et al., 2021; Sprugnoli et al., 2022). An important issue to reconcile is whether the preservation of neural circuits within tumour-infiltrated tissue implies that cognition is inevitably affected by surgeries that achieve gross total resection. Some evidence suggests that intratumoral neural activity interferes with healthy cortical processing (Aabedi et al., 2021; Krishna et al., 2023), such that removing the pathological circuitry could be expected to improve cognitive function. Such an account would seem to suggest that diffuse gliomas should be resected more radically to remove disturbing signals from the tumour. This interpretation is potentially consistent with our finding that tumour-DAN connectivity was associated with improvements in goal-directed attention after surgical resection. Alternatively, increased connectivity of the tumour with cognitive circuits may serve as a compensatory mechanism to preserve cognitive function, suggesting that more radical resection may not necessarily lead to better cognitive outcomes. The relationship between intratumoural functional connectivity and relevant clinical variables (e.g., overall survival, eloquence of tissue, and cognitive outcome) suggests that rs-fMRI could be a useful, non-invasive biomarker to help guide clinical decision making (Daniel et al., 2021; Lamichhane et al., 2021; Romero-Garcia et al., 2021). More work is also needed to determine how to best balance the amount of data required for robust, reproducible network mapping (e.g., see Gordon et al., 2017) and corresponding prediction with the time constraints of clinical scanning sessions. Future research should help delineate the biological significance of intratumoural functional connectivity to inform the interpretability of BOLD signal in neoplastic tissue.

Our findings should be interpreted within the context of some limitations which also highlight areas of future research. Because the study was originally designed to map executive function in non-lesioned cortex, concurrent ECoG-fMRI data of tumour-infiltrated tissue were available for only four of the 17 participants. While analysis of these data revealed a relationship between DAN connectivity and executive function, we also observed substantial heterogeneity in functional connectivity which likely reflects the heterogeneity of the cohort. Both the ECoG-fMRI and fMRI-only cohorts were limited by sample size (4 and 17 participants total, respectively) and heterogeneity in glioma subtype (see Table 1). Because of the limited sample size, we chose to test specific, preconceived hypotheses as opposed to comprehensively characterising the relationship between tumour functional connectivity and cognition. Future research should systematically address whether functional coupling between tumour-infiltrated tissue and healthy cortex can predict clinical outcomes. While we recruited patients suspected to have diffuse low-grade gliomas, three of these patients were found to have glioblastomas after tissue biopsy and two had gangliogliomas. Since the tumoural tissue can vary significantly across gliomas of differing molecular genetics, cellular composition and functionality, this heterogeneity could limit the generalizability of the study. Comparing tumour functional connectivity between gliomas of varying molecular genetic subtypes in future studies could enhance our understanding of the cellular basis of the interactions between tumour-infiltrated cortex and cognitive circuits.

This study provides convergent evidence from two independent modalities that cortical tissue infiltrated by diffuse gliomas participates in large-scale cognitive circuits. These findings imply functional persistence of neural circuits within glioma-infiltrated tissue, thus shedding light on mechanisms of neuroplasticity in response to neoplastic lesions. More generally, our combined ECoG-fMRI approach demonstrates the importance of cross-modality neuroimaging for advancing the understanding of functional brain networks and how they are impacted by the disease.

## CRediT authorship contribution statement

**Ayan S. Mandal:** Conceptualization, Formal analysis, Funding acquisition, Investigation, Methodology, Software, Writing – original draft, Writing – review & editing. **Chemda Wiener:** Formal analysis, Software. **Moataz Assem:** Conceptualization, Formal analysis, Methodology, Software. **Rafael Romero-Garcia:** Data curation, Formal analysis, Methodology, Software. **Pedro Coelho:** Data curation, Resources. **Alexa McDonald:** Data curation. **Emma Woodberry:** Data curation. **Robert C. Morris:** Data curation. **Stephen J. Price:** Conceptualization, Investigation, Supervision. **John Duncan:** Conceptualization, Supervision. **Thomas Santarius:** Conceptualization, Data curation, Investigation, Supervision. **John Suckling:** Conceptualization, Funding acquisition, Supervision. **Michael G. Hart:** Conceptualization, Data curation, Formal analysis, Funding acquisition, Investigation, Methodology, Supervision. **Yaara Erez:** Conceptualization, Data curation, Formal analysis, Funding acquisition, Investigation, Methodology, Software, Supervision, Writing – original draft, Writing – review & editing.
