# Supplementary material for: Tumour-infiltrated cortex participates in large-scale cognitive circuits
Source: Cortex. 2024 Apr;173:1–15. doi: 10.1016/j.cortex.2024.01.004 (PMC10988771; doi:10.1016/j.cortex.2024.01.004)
Supplement: Multimedia component 1 [file mmc1.docx]

Tumour-infiltrated cortex participates in large-scale cognitive circuits

Supplementary Materials

Ayan S. Mandal^1,2,^ Chemda Wiener^3^, Moataz Assem^4^, Rafael Romero-Garcia^2,5^, Pedro Coelho^6^, Alexa McDonald^7^, Emma Woodberry^7^, Robert C. Morris^7^, Stephen J. Price^8^, John Duncan^4,10^, Thomas Santarius^8,9,11^, John Suckling^2,12,13^, Michael G. Hart^8,14^, Yaara Erez^3,4,15^

1 Brain-Gene Development Lab, Department of Psychiatry, Perelman School of Medicine at the University of Pennsylvania

2 Brain Mapping Unit, Department of Psychiatry, University of Cambridge

3 Faculty of Engineering, Bar-Ilan University, Ramat-Gan, Israel

4 Medical Research Council, Cognition and Brain Sciences Unit, University of Cambridge

5 Department of Medical Physiology and Biophysics, Instituto de Biomedicina de Sevilla (IBiS) HUVR/CSIC/Universidad de Sevilla/CIBERSAM, ISCIII, Sevilla, Spain

6 Neurophys Limited

7 Department of Neuropsychology, Cambridge University Hospitals NHS Foundation Trust

8 Department of Neurosurgery, Cambridge University Hospitals NHS Foundation Trust

9 Division of Neurosurgery, Department of Clinical Neurosciences, University of Cambridge.

10 Department of Experimental Psychology, University of Oxford

11 Department of Physiology, Development and Neuroscience, University of Cambridge

12 Behavioural and Clinical Neuroscience Institute, University of Cambridge

13 Cambridge and Peterborough NHS Foundation Trust

14 Neurosciences Research Centre, Institute of Molecular and Clinical Sciences, St George’s, University of London & St George's University Hospitals NHS Foundation Trust

15 Gonda Multidisciplinary Brain Research Center, Bar-Ilan University, Ramat-Gan, Israel

Correspondence to:

Ayan S Mandal, 3400 Civic Center Blvd, Philadelphia, PA 19104 Ayan.Mandal@pennmedicine.upenn.edu

Yaara Erez, Bar-Ilan University, Ramat Gan, Israel 5290002 yaara.erez@biu.ac.ul

**Supplementary Figure 1. Placement of electrodes within tumour-infiltrated tissue.** Electrode locations are represented in red. The outline of each tumour mask is coloured green.


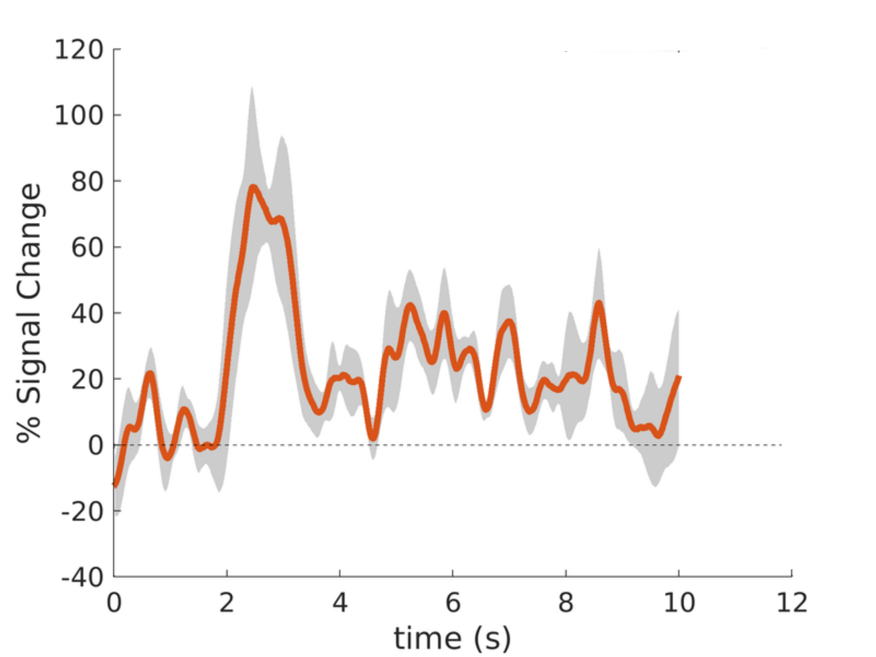


**Supplementary Figure 2. Time course of high gamma power modulations in tumour-infiltrated tissue for the hard vs. easy contrast**. Average time course across all electrodes that showed significant change in power for this contrast, smoothed by a 1000 ms Gaussian kernel (SD=200 ms). Data is average across all trials, cropped to the shortest trial duration. Shaded area indicates SEM.

**Supplementary Figure 3. The relationship between tumour-DAN connectivity and performance on the Hearts Cancellation task stratified by glioma subtype.** Scatterplots of the relationship between overall accuracy on the Hearts Cancellation Task and tumour-DAN connectivity at the preop and latest follow up timepoints, as well as the difference between the follow up and preoperative scores stratified by glioma subtypes indicated in Table 1. The preoperative and follow-up Heart Cancellation Scores were Z-score normalized using descriptive statistics from healthy participants.

| Network | Delta  (1-4 Hz) | Alpha  (8-12 Hz) | Beta  (12–30 Hz) | Gamma  (30-70 Hz) | High Gamma (70-250 Hz) |
| --- | --- | --- | --- | --- | --- |
| VN | 0.99 (0.32) | 4.12 (NA) | 14.1 (NA) | 1.7 (0.19) | 1.3 (0.25) |
| SMN | 1.9 (0.17) | 0.24 (0.24) | **7.7 (0.0055)** | 1.0 (0.31) | 0.88 (0.35) |
| DAN | **4.8 (0.028)** | 0.34 (NA) | 3.7 (NA) | **6.2 (0.013)** | **4.3 (0.038)** |
| VAN | 5.4 (NA) | 0.54 (0.37) | 3.3 (0.069) | 1.5 (0.23) | 0.15 (0.70) |
| LIM | 0.31 (0.58) | 0.012 (0.91) | 0.16 (0.69) | 0.34 (0.56) | 2.4 (0.12) |
| FPN | 1.3 (0.25) | 1.4 (NA) | 4.2 (NA) | 0.98 (0.32) | 1.1 (0.30) |
| DMN | 0.51 (0.47) | 0.078 (0.78) | 0.35 (0.56) | 0.23 (0.63) | 1.40 (0.24) |

**Supplementary Table 1. Relationship between functional network connectivity and power modulations for hard>easy contrast across different frequency bands.** The relationships between functional network connectivity and power modulations were assessed using linear mixed-effects models. Chi-square test statistics are displayed with the corresponding *P*-values in parentheses. In certain cases, a *P*-value could not be accurately calculated because of a poorly fit model, indicated in the table as “NA”.

| Predictor | Standardized Estimate | Standard Error | T Statistic | *P* Value |
| --- | --- | --- | --- | --- |
| *Response variable = Overall Hearts Cancellation Accuracy (follow-up)* (n = 13) | | | | |
| Intercept | NA | 0.435 | -1.7 | NA |
| Pre-op score | 0.20 | 0.11 | 1.3 | 0.28 |
| Tumour-DAN Connectivity | **1.37** | **0.77** | **6.2** | **0.0035** |
| Location (insula) | -0.57 | 0.34 | -1.0 | 0.39 |
| Location (temporal) | **0.78** | **0.18** | **4.8** | **0.0086** |
| Hemisphere (right) | -0.20 | 0.21 | -0.96 | 0.39 |
| Latest Assessment (Month 3) | 0.15 | 0.17 | 0.95 | 0.39 |
| Age | 0.013 | 0.0078 | 0.071 | 0.95 |
| Gender (male) | 0.011 | 0.15 | 0.068 | 0.95 |
|  |  |  |  |  |

**Supplementary Table 2. Results of multiple linear regression models predicting Tumour-DAN Connectivity and long-term outcomes on the Heart Cancellation task, excluding participants without a Month 3 or greater follow-up assessment.**
